# Effect of a Combined Antidepressant Drug Flupentixol–Melitracen on Glucose Level and Expression of Insulin‐Like Peptide Genes *DILP5* and *DILP6* in *Drosophila melanogaster*

**DOI:** 10.1155/bmri/6698526

**Published:** 2026-04-30

**Authors:** Azizul Islam Barkat, Khandaker Asif Ahmed, Sumaiya Akter, Md Shamsudduha, Farhin Momtaz Riana, Rabeya Bosri Mehe Jabin, Mahmuda Begum, Mohammad Shamimul Alam

**Affiliations:** ^1^ Genetics and Molecular Biology Laboratory, Department of Zoology, University of Dhaka, Dhaka, Bangladesh, du.ac.bd; ^2^ CSIRO Agriculture and Food, Canberra, Australian Capital Territory, Australia, csiro.au; ^3^ Zoology Section, Biological Research Division, Bangladesh Council of Scientific and Industrial Research (BCSIR), Dhaka, Bangladesh

**Keywords:** bioinformatics, depression, frenxit, insulin, LC_50_

## Abstract

The number of depression sufferers is increasing at an alarming rate around the world. As a result, the usage of antidepressant drugs is growing day by day. However, the effect of this medication on glucose homeostasis is not clear. We investigated the impact of a combined antidepressant drug flupentixol‐melitracen on glucose levels and the expression of associated genes in *Drosophila melanogaster*. The flies were reared in control and treatment vials having standard and drug‐treated food. The glucose oxidase method showed significantly reduced glucose levels in the treated *Drosophila*. The Ct values obtained from qPCR for the relative quantification of *Drosophila* insulin‐like peptide (DILP) genes, *DILP5* and *DILP6*, were analyzed using the 2^−*ΔΔ*CT^ method. The *DILP5* gene was expressed slightly higher in the antidepressant‐treated group, though the expressions of the DILP genes appeared statistically insignificant. The current study suggests that anxiolytics and antidepressant drugs might be associated with the insulin signaling pathway, which is crucial for growth, glucose regulation, and other fundamental metabolic processes. Further study is required to determine the expression pattern of all DILP and other genes that might affect glucose homeostasis.

## 1. Introduction

Depression is one of the major mental disorders worldwide and become a public health issue globally. The problem is characterized by despair, pessimism, and losing interest in once‐enjoyed pursuits. People of any age can suffer from the problem, resulting in difficult life circumstances, hormone imbalances, and genetic predisposition [1]. The prevalence of individuals suffering from depression is increasing rapidly. The number of depression cases has increased by 49.86% in about three decades estimated, from 172 million in 1990 to 25.8 million in 2017 [2]. Depression and anxiety frequently coexist, with individuals experiencing one condition often exhibiting symptoms of the other [3].

Antidepressant drugs are commonly prescribed medications used to treat various mental health conditions, primarily depression and anxiety disorders. However, the rapid usage of these drugs poses many side effects, including nausea, digestive problems, sexual dysfunction, weight gain or weight loss, and sleep disturbances. Despite having many side effects, the usage of the medicines is increasing day by day [4]. It operates by altering the proportion of particular brain chemicals that regulate mood, such as serotonin and norepinephrine [5]. Antidepressants are available in different forms, including tricyclic antidepressants, monoamine oxidase inhibitors, selective serotonin reuptake inhibitors (SSRIs), and serotonin–norepinephrine reuptake inhibitors. Each of these medicine types operates somewhat differently and may vary in effectiveness for different individuals [6]. Although SSRIs are commonly considered first‐line antidepressants, flupentixol–melitracen has been shown to be effective in patients with mild‐to‐moderate depression and anxiety. Some evidence suggests this drug combination tends to relieve symptoms of depression or anxiety more quickly than many common antidepressants, such as SSRIs [7]. Flupentixol achieves its effects by blocking the activity of dopamine D2 receptors, limiting dopamine activity in the brain. Dopamine regulates the sensations of pleasure, motivation, and mood. The reduction of dopamine leads to psychotic symptoms such as hallucinations and delusions [8]. Flupentixol also activates serotonin 5‐HT2 receptors, contributing to its antidepressant effects [9]. Melitracen blocks the reuptake of serotonin and norepinephrine in presynaptic terminals in neurons [10, 11]. Serotonin plays a regulatory role in insulin secretion by triggering the signaling cascade of the PI3K‐Akt‐TOR pathway [12, 13]. Moreover, it may affect the expression or sensitivity of glucose transporters and receptors on target tissues influencing cellular glucose uptake and overall glucose homeostasis [14]. The combination of flupentixol (0.5 mg) and melitracen (10 mg) is anxiolytics and antidepressant drugs which are often prescribed for mixed anxiety‐depressive states and psychosomatic disorders, especially when rapid mood stabilization is desired or when patients show limited response to SSRIs [7, 11]. While its clinical efficacy is well recognized, little is known about how this drug combination influences metabolic processes, including glucose regulation.

*Drosophila*, a potential model for depression research [15], has been extensively used in scientific research as a model organism for various fields, including genetics, physiology, and neurobiology [16, 17]. In *Drosophila*, IPCs (insulin‐producing cells) are similar to vertebrate pancreatic *β*‐cells secreting insulin in response to hyperglycemia. At the same time, corpus cardiacum (CC) serves the purpose of *α*‐cells to produce glucagon‐like hormones. Due to the similarity of their functions to those of *α*‐ and *β*‐cells, IPCs and CC, taken together, are seen as the *Drosophila* analog of the mammalian pancreatic gland [18].

*Drosophila* insulin‐like peptides (DILPs) are proteins that share strong evolutionary and functional similarity to the human insulin found in *Drosophila* and other insects. Eight DILP genes (*DILP1–8*) have been identified from *Drosophila* species [19–21]. Seven of these peptides (*DILP1–7*) bind the *Drosophila* insulin receptor (dInR), whereas *DILP8* binds the leucine‐rich repeat‐containing G protein‐coupled receptor 3 (Lgr3). Upon interaction with specific receptor, these peptides maintain a balance between the stored and circulating carbohydrates triggering signaling cascades. They also contribute to other function regarding development and reproduction [20]. The expression patterns of these eight genes are different and respond to varying conditions in various cells [20, 21]. Intriguingly, these genes show redundancy in function [21]. The function of *DILP*s′ knockout mutant is maintained by other DILPs. Mutant *Drosophila*, consisting of knockout genes that code for DILPs, shows various growth defects. Moreover, removing IPCs creates a phenotype mimicking human Type 1 diabetes [22]. Additionally, several studies have demonstrated that serotonergic signaling modulates IPCs in *Drosophila*, which has orthologs in mammals as evident by FlyBase [23–26]. Some epidemiological studies suggest that individuals, suffering with depression are at a higher risk of developing Type 2 diabetes [27]. Specifically, those with depression have a 60% increased risk of developing the disease [28]. Morever, these two conditions share a bidirectional relationship wherein the presence of one significantly elevates the risk of developing the other [29, 30]. Depression contributes to this risk through behavioral factors like reduced physical activity, poor diet, sleep disturbances, and medication nonadherence, which negatively impact metabolic health [31, 32]. Despite this established link, the effect of antidepressant pharmacotherapy on glucose homeostasis is unclear.

In this study, two representative genes, *DILP5* and *DILP6*, were selected from eight genes showing similarity to mammalian insulin‐regulating glucose homeostasis genes [21, 28, 29]. We investigated how the antidepressant combination flupentixol–melitracen affects glucose levels and associated insulin‐related gene expression in *Drosophila melanogaster*. Using this model, we seek to identify possible connections between antidepressant treatment and glucose regulation, helping to better understand how such drugs may influence metabolism.

## 2. Experimental Procedures

### 2.1. Measurement of Apical End Points and Corresponding Glucose Concentration

For this experiment, we collected wild *Drosophila* from the Zoological Garden, University of Dhaka in June 2023, using banana baits. The flies were reared in semolina‐yeast food medium and maintained in a 3‐week lifecycle. For egg‐laying, the food vials were replaced every 3–4 days. Each food vial contained 20–25 flies. All the maintenance was conducted in a confined room at 25^°^C ± 1^°^C temperature, 65% relative humidity, and in a 12:12 light–dark cycle. The third instar larvae were used to measure apical end points for the antidepressant drug.

The antidepressant drug, Frenxit (a combination of 0.5 mg flupentixol and 10 mg melitracen), manufactured by Beximco Pharmaceuticals Ltd. (Bangladesh), was obtained from a local pharmacy and ground to ensure complete dissolution in distilled water. The outer pigmented layer of the pill was stripped away from the drug, and weight was measured to determine the lethal concentration, LC_50_, and the drug was crushed and mixed with a 5% sugar–water solution. To measure the apical end points, six treatment concentrations, that is, 100, 200, 300, 400, 500, and 600 mg/L were considered. A control group (0 mg/L) was also taken into account. Then, 15 third instar larvae were transferred to each treatment and control vials and observed for 24 h. After treatment, the larvae were recovered, and the number of survivors was recorded and analyzed to identify the end point. Six biological replicates were considered for this assay.

The collected data was analyzed to determine LC_50_. We evaluated the LC_50_ dose to assess maximal pharmacological stress without causing excessive toxicity [33]. The desired dose, identified from this assay, was used as the only treatment group in subsequent glucose concentration measurement.

For glucose concentration measurement, we modified the standard food media, as described in a previous study [34]. It involved adding desired concentrations of drug at the last stage of cooking, which allowed us to make a homogeneous mixture before solidification. For control flies, flies were maintained in standard media. The control group was treated with distilled water in the same volume as used for the treatments. A total of 15 vials (biological replicates), each containing 30 ± 2 flies, were considered for each of the treatment and control groups. The adult flies were kept in the vials for 48 h to facilitate egg deposition, and subsequently, the adults were discarded. After hatching, the larvae continued to feed and develop on either the treated medium or the control food. As the larvae reached the third instar larval stage (96 h), they were harvested and subjected to glucose level measurement. The third instar larvae were chosen as they represent the most metabolically active developmental stage in *Drosophila*, consistent with previous studies on insulin signaling pathways [35, 36]. From each vial, a pool of three third instar larvae was transferred into a 1.5‐mL microfuge tube, containing 50 *μ*L PBS, and squashed to get the whole‐body homogenates. After squashing, the tubes were centrifuged at 13,000 rpm for 1 min to precipitate cellular debris. Then, 10‐*μ*L tissue homogenates were used for the colorimetry test to detect glucose levels. Then, 20 biological replicates were taken for the experiment. The glucose concentration was measured using the Glucose MR kit (Linear Chemicals, Spain) [37]. The absorbance was measured in a spectrophotometer (Spectrophotometer 721, China) at 500‐nm wavelength for analyzing glucose levels in the control and treatment groups.

### 2.2. Identification of Human Glucose–Related Genes and Their Orthologs in *Drosophila*

Our literature search has identified several glucose‐ and insulin‐related genes. We conducted a blast and reciprocal blast search in insect model, *D. melanogaster* genome, and identified *DILP* genes are structurally and functionally closely related to human glucose– and insulin‐related genes. We revisited the modENCODE: *Drosophila* transcriptome, developmental stage dataset, and narrow down our focus for only two *DILP* genes, which showed consistent expression patterns, across all larval stages. To study phylogeny and structural similarities, longest protein sequences of 8 DILP genes and their human orthologs (*IGF1*, *IGF2*, and *INSL5*) were extracted from flybase and NCBI, respectively, aligned using MAFFT v7.2.1.5 [38]. The aligned fasta file was used in IQ‐Tree [39], which conducted selected optimal statistical model, ML‐based phylogeny, and bootstrap analysis using its default 1000 bootstrap replicates. The tree was visualized using iTol web server v. 6.9.1 [40].

For structural similarity analysis, the aligned file was used as input in MEME web server [41] (accessed on 9 September 2024) and then ran in classic mode with the number of motifs set to five. The identified motifs were named and numbered based on their similarities and positions. For conserved domain prediction, protein sequences were searched against NCBI curated domains (containing 19,902 position‐specific scoring matrices) of NCBI conserved domain database [42–44] with *e* value < 0.01 and a maximum number of hits of 500.

### 2.3. Gene Expression Analysis for Two DILP Genes

As gene expression study is sensitive, we considered the Hikone‐H strain of *D. melanogaste*r (DGRC Number: 109‐146), which is an isofemale strain and well adopted in sensitive laboratory assays. The flies were maintained in identical laboratory conditions, as described above. Similar experimental settings of one treatment and one control group were utilized. Total RNA was extracted from a pool of 10 third instar larvae using a TRIzol‐based RNA extraction kit (Tiangen, China). Three biological replicates were considered for each of the control and treatment groups. The extracted RNA was immediately converted into cDNA using the First Standard Synthesis Kit (Abclonal, United States). Then, 20 *μ*L of cDNA was diluted four times with nuclease‐free water and the diluted cDNA was used in the downstream qPCR assay.

The expression of two DILP genes was analyzed using a dye‐based reagent (TaKaRa TB Green, China) in a qPCR machine (Analytic Jena: qTOWER^3^G). The ribosomal protein *RPL11* was used as a housekeeping gene in this study. The expression of these two genes was analyzed from three controls and three treatment cDNAs (three biological replicates). Each reaction was conducted in triplicate. The primer sets used in this study are given in Table 1. The thermocycle condition includes 5 min at 95°C for initial denaturation, 30 s at 95°C for denaturation, 30 s at 60°C for annealing, 20 s at 60°C for extension, and 15 s for final elongation. The steps were run 35 times. The relative expression of the targeted genes was analyzed by the 2^−*ΔΔ*CT^ method [45].

**Table 1:** Primer sequences used for qPCR experiment.

**Gene name**	**Forward sequence (5** ^′^ **-3** ^′^ **)**	**Reverse sequence (5** ^′^ **-3** ^′^ **)**
*RPL11*	AACTTCGGTTTCGGCATCCA	TTGCGCTTCCTGTGGTTCA
*DIPL5*	TGTTCGCCAAACGAGGCACCTT	CACGATTTGCGGCAACAGGAGTCG
*DILP6*	TGCTAGTCCTGGCCACCTTGTTCG	GGAAATACATCGCAAGGGCCACC

### 2.4. Statistical Analysis

All visuals and statistical analyses were carried out in R Studio (Version 2024.04.0), under the ggplot2 package [46]. To estimate mean survival rate at different dose responses, a linear regression model was fitted to quantify the relationship between antidepressant dose concentrations (milligrams per liter) and mean larval survival. Model parameters, including slope, intercept, and coefficient of determination (*R* ^2^), were highlighted. The LC₅₀ was estimated as the concentration corresponding to 50% survival.

To compare measurements of glucose concentration between control and treatment, a normality was assessed using the Shapiro–Wilk test (by shapiro.test() function). As the concentrations did not follow a normal distribution (*p* < 0.05), a nonparametric Wilcoxon rank‐sum test (Mann–Whitney *U* test) was used to compare groups. Similarly, to assess raw Ct values and relative expression patterns of *DILP5* and *DILP6* genes, between control and treated larvae, a normality test was evaluated using Shapiro–Wilk test, followed by Wilcoxon rank‐sum testing. For all cases, statistical significance was defined as *p* < 0.05.

The visualizations were produced using ggplot2 (v.3.5.2) and ggpubr (v. 0.6.1) R‐packages.

## 3. Result

### 3.1. Apical End Point in Survivability

**Figure 1:**
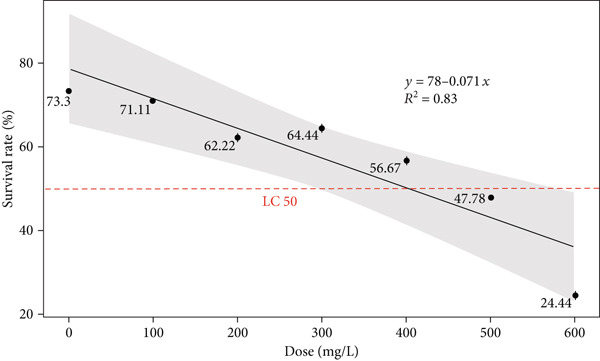
Survival rate of *Drosophila* larvae at six different doses of antidepressant drug. Mean survival rate (%) with standard error bars is shown. LC_50_ is shown with red horizontal bar. The best‐fit linear regression line, slope‐intercept, and *R* ^2^ values are also shown.

A lethal concentration of 50, that is, LC_50_ dose of the antidepressant drug for *Drosophila* third larvae was determined by applying the drug at several concentrations. The datapoints of Figure 1 showed observed survival rates, with some variations around the regression line shown by the shaded confidence interval (Figure 1). Overall, our result showed a strong negative correlation between survival rate and antidepressant drug doses (*y* = 78 − 0.071*x*, *R* ^2^ = 0.83). Under the current experimental scenario, the survival rate (*y*) starts at 78% and for every unit of dose (milligrams per liter), the survival rate decreased by 0.0071%. The survival rate was approximately 50% at around 400 mg/L, where the trend line intersects the horizontal red line (representing LC_50_), while the survival rate at 500 and 600 mg/L was 47.78% and 24.44%, respectively. The dose used in subsequent study was 400 mg/L which is lower than LC_50_.

### 3.2. Glucose Concentration Measurement

**Figure 2:**
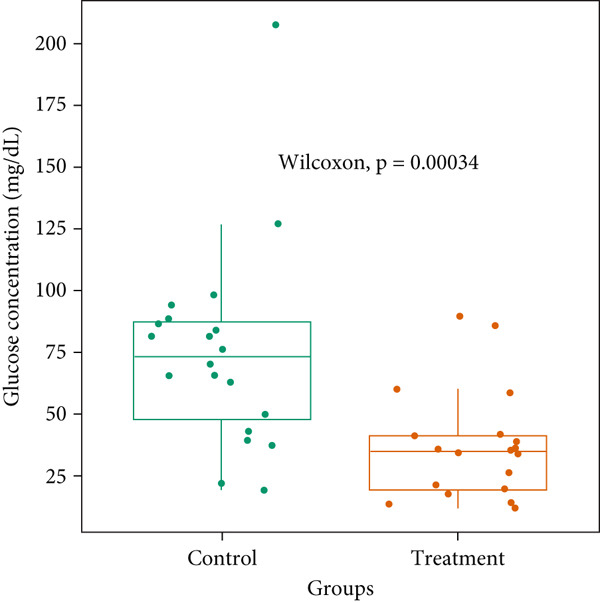
Glucose concentration at the control and treatment groups. Tissue homogenates of 20 biological replicates for each of the control and treatment groups were considered. The concentration was measured by a spectrophotometer at 500‐nm wavelength. The Wilcoxon test showed significant variance (*p* < 0.005) between mean values of the control and treatment groups.

Third instar larvae from the control and treatment groups were taken to measure glucose levels. The mean glucose concentrations of the control and treatment groups were 75.02 (± 40.13) and 36.54 (± 21.79), respectively. A nonparametric Wilcoxon test showed low *p* values (*p* < 0.005), which indicated a statistically significant difference in glucose concentrations between both groups. Our result suggested that the treatment group (at 400 mg/L) had a reduced glucose concentration relative to that of control. Further, we observed a wider interquartile range (IQR) and more spread‐out datapoints in the control group **(**Figure 2 **)**, indicating heterogeneity of datapoints with greater variability of glucose concentrations in control replicates compared to treatment. The treatment group has a narrower IQR, suggesting more consistency in glucose concentrations. The absence of extreme outliers and narrow variability in the treatment group implies that the treatment could effectively reduce and stabilize glucose levels.

### 3.3. Selection of Human Insulin–Like Gene Orthologs in *D. melanogaster*

Our comprehensive bioinformatics and literature search has identified several orthologs of important human insulin–like genes in *D. melanogaster*. We have identified several DILP genes, which showed strong orthology with at least one of the human insulin growth factors (*IGF1* and *IGF2)* or insulin (*INSL5)* genes. For example, *DILP1–3*, *DILP5*, and *DILP6* showed strong homology with both human *IGF1* and *IGF2* genes. *DILP4* showed similarity with the human insulin gene (INSL) only. No human ortholog was detected for *DILP7* and *DILP8.* Such discrepancy warns us to proceed further with all the DILP genes for subsequent expression analysis.

**Figure 3:**
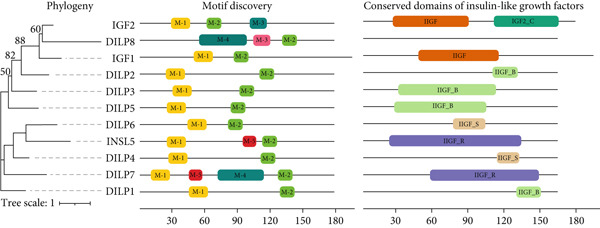
Phylogeny, conserved motifs, and domains among eight *DILP* genes (*DILP1–8*) and their human orthologs (*IGF1*, *IGF2*, and *INSL5*). Motif discovery showing conserved motifs across all 11 sequences, with *e* < 0.01, *w* *i* *d* *t* *h* > 10 cut‐off. Conserved domain search was conducted against NCBI CDD database. IIGF refers to insulin‐like growth factor, IIGF_B refers to insulin‐like growth factor bombyxin_like subgroup, IIGF_S refers to insulin‐like growth factor superfamily, and IIGF_R refers to insulin‐like growth factor relaxin like.

Phylogeny and structural similarities among the products of these genes gave insights to narrow down our focus. The phylogenetic tree depicts two major clusters, *DILP4*, *DILP6*, and *DILP7* cluster together with human *INSL5* protein, whereas the rest of the DILP protein, except *DILP1* protein, cluster with human *IGF1* and I*GF2* (Figure 3). Even though *DILP8* formed a cluster with human IGF, the longer branch length showed diversification of the protein. Our motif discovery plot showed the existence of five distinct motifs across the 11 proteins studied. The size of motifs varied from 13aa (M‐5) to 44aa (M‐4). M‐1 (14aa) and M‐2 (19aa) motifs appeared across all *Drosophila* and human proteins, indicating their evolutionary conservation and might play fundamental roles in the structure and function of both organisms. Further, some distinct motifs were also detected, for example M‐5 motif in *DILP5* and *INSL5*; M‐3 (17aa) motif in *IGF2* and *DILP8*, and M‐4 in *DILP7* and *DILP8*—which indicate their association with specialized functions or lineage‐specific adaptations to maintain functional integrity.

Our conserved domain search has identified several conserved protein domains of insulin growth–like superfamily. The most common domain was IlGF_B (insulin‐like growth factor, bombyxin like), a peptide hormone in insects, appearing in *DILP1*, *2*, *3*, and *5.* Species‐specific appearance of distinct domains was also observed. For example, IlGF_S (insulin‐like growth factor superfamily) in *Drosophila* DILP genes (*DILP4* and *DILP6*) and IlGF_F (insulin‐like growth factor) only appeared in human IGF genes (*IGF1* and *IGF2).* Further, IGF2_C (insulin‐like growth factor II E‐peptide), found in C‐terminal domain of vertebrate *IGF2* gene only, observed only in human *IGF2* gene, but not in any other human IGF or *Drosophila* DILP genes, highlighting functional conservation of this domain. Further, IlGF_R (insulin‐like growth factor, relaxin like), a family specific to vertebrates appeared in not only in human *INSL5*, but also in *Drosophila DILP5* genes, showing functional conservation of this domain in model insects as well. It is noteworthy that no IlGF domain was detected for *DILP8* gene, further validating our robust ortholog assignment criteria. Depending on our comparative analysis, both *DILP5* and *DILP6* genes showed well representation of human insulin–like genes, and we selected these two genes for downstream qPCR assay.

**Figure 4:**
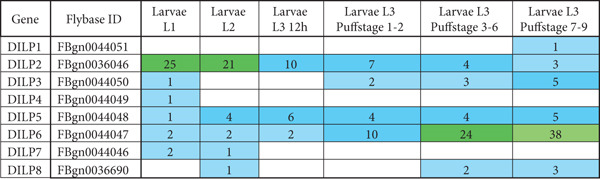
RKPM values (reads per kilobase per million mapped reads) for different DILP genes at different larval stages. The values are adopted from modENCODE: *Drosophila* transcriptome, developmental stage dataset. The cells are color‐coded (< 3: light blue; 4–10: dark blue; 11–25: dark green; > 26: light green), based on flybase expression level color levels. Empty cells indicate no/extremely low expression.

Further, we revisited the modENCODE: *Drosophila* transcriptome dataset to investigate the expression patterns of *DILP* genes. Among the insulin‐like peptide genes in *Drosophila*, the expressions of *DILP5* and *DILP6* are consistent in all three larval stages (Figure 4) though distinct spatiotemporally. Expression analyses show that both genes are active throughout various developmental stages and tissues, including the nervous and digestive systems. Even though the expression of the *DILP2* gene is evident across all larval stages, it starts to disseminate over time. So, the expression profile of DILP genes further indicates the use of *DILP5* and *DILP6* genes for the expression study.

### 3.4. Gene Expression Analysis

**Figure 5:**
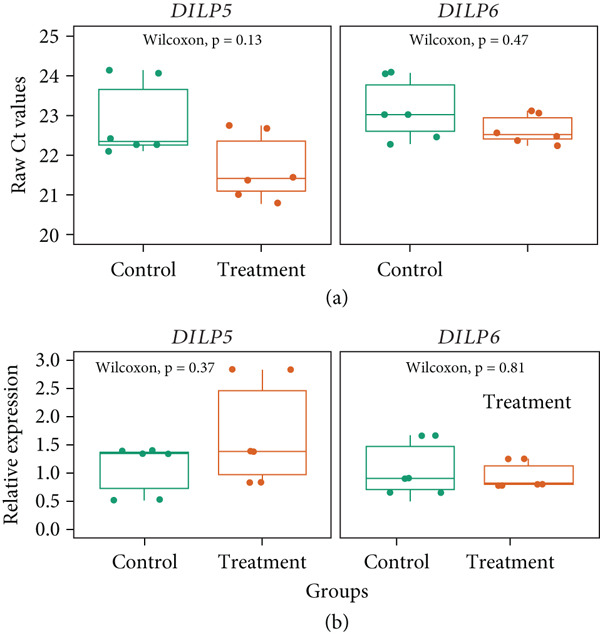
Gene expression of *DILP5* and *DILP6* genes. Boxplots show (a) raw Ct values and (b) relative gene expression of *DILP5* and *DILP6* genes. Wilcoxon test between the means of the control and treatment groups showed no significance in any comparison.

Our gene expression study involved initial bioassay involving treatment (treated with 400 mg/L Frenxit) and control groups of isofemale Hikon‐H strain of *D. melanogaster*. We conducted a Sybr‐green–based qPCR assay, targeting *DILP5* and *DILP6* genes, including housekeeping gene. Overall, for both *DILP5* and *DILP6* genes, we did not find any significant difference in raw Ct values between the treatment and control groups (with *p* values of 0.13 and 0.47, respectively, with Wilcoxon tests) **(**Figure 5a**)**. The distribution of the Ct values showed minimal variabilities within groups, reflecting uniform amplification efficiency and reliability of qPCR measurements.

Similarly, we could not detect any significant difference in the relative expression of these two genes in both tested groups (Figure 5b). The *DILP5* gene expression was slightly higher in the treatment group (1.69 ± 1.03), compared to the control groups (1.09 ± 0.49) **(**Figure 5b**)**, but a high *p* value (*p* = 0.37) indicates no significant differences between the groups. On the other hand, a slight decrease in relative expression of the *DILP6* gene was observed in the treatment group (0.95 ± 0.26), compared to the control (1.08 ± 0.52), but still missed the significance (*p* = 0.72). Overall, our analysis indicates that neither *DILP5* nor *DILP6* expression is significantly altered by the treatment, as evidenced by both raw Ct values and relative expression data.

## 4. Discussion

Chronic stress and prolonged exposure to depression can disrupt insulin signaling pathways, leading to insulin resistance and impaired glucose homeostasis. Such metabolic disturbances are key risk factors associated with the development of obesity and Type 2 diabetes [47–50]. However, the role of flupentixol–melitracen, a combined antidepressant utilized for the treatment of depression and other mental health conditions, on glucose homeostasis remains unclear. Exploring the mechanisms by which these medications affect metabolic pathways is essential for understanding their potential side effects. This study assessed the effect of these drugs on glucose metabolism and the expression of two *DILP* genes, *DILP5* and *DILP6*, in *D. melanogaster*.

### 4.1. Antidepressant Combination Flupentixol–Melitracen Reduces Glucose Amount in Vinegar Flies

In humans, antidepressants have been shown to improve glucose homeostasis and insulin sensitivity in Type 2 diabetes (T2D) patients by alleviating coexisting depression. Prolonged antidepressant use has been associated with hyperglycemia and an increased risk of T2D in nondiabetic individuals [51, 52]. The present study indicates that the drug causes a significant decrease in the glucose level of *Drosophila* larvae. In *Drosophila*, the principal circulating sugar in hemolymph is trehalose rather than glucose. Glucose from diet and digested food materials in the gut is converted into trehalose in the fat body. The fat body of an insect serves like mammalian liver adipose tissue [53, 54]. The effect of the Frenxit drug on trehalose levels could be worthy of research. In humans, the primary circulating carbohydrate is glucose. The current study indicates that the antidepressant Frenxit drug significantly alters glucose levels, more specifically, by decreasing glucose levels. Insulin‐like peptides are secreted in insects when there is adequate glucose to promote more glucose uptake in cells. Its secretion is reduced when glucose is inadequate in the hemolymph, resulting in decreased uptake. The activity of insulin‐like peptides is similar to that of mammalian insulin [20].

Stress and negative environmental factors have been shown to elevate glucose levels in *Drosophila* [65] and disrupt glucose homeostasis in humans, potentially leading to Type 2 diabetes [47]. Clinical studies also highlight a significant association between antidepressant use and hyperglycemia in humans [66]. Interestingly, stressful conditions are linked to developing a Type 2 diabetes–like phenotype in *Drosophila* [65] and humans [47]. In this study, antidepressant treatment appeared to lower glucose levels, probably by stimulating insulin production. This results in *Drosophila* being insightful for humans. Glucose and trehalose levels in high‐sugar diets increase significantly and grow insulin‐resistant or Type 2 diabetes phenotype in *Drosophila* [55]. Another study indicates that the Type 2 diabetic *Drosophila* produced from the high sugar diet consists of high trehalose and low expression of the *DILP5* gene [56]. High‐sugar diet, stress or depression, and antidepressants all affect carbohydrate metabolism.

### 4.2. Orthologs of Human Glucose– and Insulin‐Related Genes

Antidepressants cause significant changes in the expression of many genes [57]. In a genome‐wide association study, major depressive disorder and antidepressant‐treated patients showed significant changes in gene expression [57]. However, the effect of the drugs on glucose homeostasis genes is not clear. These DILPs are analogs of mammalian insulin, which regulates glucose metabolism, growth, development, stress response, and other functions. There are conserved domains among the DILP genes in *Drosophila* (Figure 3). *DILP5* and *DILP6* reportedly involve insulin receptor binding function and carbohydrate homeostasis [19, 60]. *DILP6* is also related to starvation response [60, 63‐64]. Therefore, studying the *DILP5* and *DILP6* genes of *Drosophila* which show the highest similarity to mammalian insulin–regulating glucose homeostasis genes could be valuable [21, 58, 59].

### 4.3. *DILP5* and *DILP6* Genes Lack Major Role in Antidepressant Drug–Dependent Glucose Reduction

We found insignificant changes in glucose‐regulating genes, *DILP5* and *DILP6*, in this study. Thus, the role of *DILP5* and *DILP6* in glucose level reduction could not be ascertained in *Drosophila*. Though *DILP5* gene has shown somewhat higher expression level in the treated group, it was not statistically significant. However, in mouse models, *DILP5* has been proven to bind with human insulin receptors and lower the glucose level [60]. The expression pattern of *DILP6* in this study′s control and treatment groups is similar, probably due to the third instar larvae, which have a low expression. *DILP6* is expressed at a low rate in the gut [19] and has been proven to be expressed highly in the fat body during the transition from larval to pupal stages and in the pupal stage. Therefore, to investigate the proper effect of the drug on *DILP6* gene expression, pupae should be included in further studies. The discussion forwards that *DILP5* and *DILP6* might not have a major role in antidepressant drug–dependent glucose reduction in *Drosophila*. Side by side, since there are some variances in gene expression, any secondary impact of these genes on glucose homeostasis cannot be ruled out. Conducting a comprehensive study on all DILP genes could be valuable. Additionally, the acquisition and analysis of transcriptomic data of the treated and control groups might help investigate the molecular basis of this phenomena.

### 4.4. Serotonergic Modulation of Insulin Signaling in *Drosophila*

The combination of flupentixol and melitracen exposure may affect glucose metabolism in *Drosophila* through serotonergic regulation of insulin‐like peptides. The drug, Fenxit, modifies serotonin, which is known to influence the activity of IPCs in the fly brain [23, 24]. These IPCs secrete insulin‐like peptides that bind to the dInR in peripheral tissues. Activation of dInR triggers the canonical PI3K‐Akt‐TOR pathway, regulating carbohydrate metabolism, glucose/trehalose transporter expression, and glycogen storage [61, 62]. Therefore, alterations in serotonergic signaling induced by flupentixol–melitracen may enhance DILP secretion, activate insulin receptors, and subsequently promote glucose metabolism, ultimately resulting in reduced glucose concentration. This mechanistic link provides a hypothetical explanation for the observed changes in glucose concentration and DILP gene expression following Frenxit, the combined antidepressant drugs′ exposure in this study.

## 5. Conclusion

The usages of combined drugs flupentixol–melitracen cause significant changes in the glucose level of *Drosophila*. The *DILP5* expression is affected slightly by these drugs. However, the expression of *DILP6* appears to be unchanged. The present study suggests that antidepressant drugs may affect the insulin signaling pathway, the primary path for growth, glucose homeostasis, and other basic metabolisms. Further study is required to investigate the expression and function of eight DILP genes. Additionally, it can provide crucial insight into antidepressant medication for depression, anxiety, and Type 2 diabetic patients.

## Conflicts of Interest

The authors declare no conflicts of interest.

## Funding

This work was partially funded by the University Grants Commission of Bangladesh, Biological Science 2020‐21 (Received by M.S.A. as Principal Investigator), and Ministry of Science and Technology, Government of the People′s Republic of Bangladesh, (through National Science and Technology Fellowship received by A.I.B.).

## Data Availability

The data will be available upon request.
